# Comparison of proximate composition and sensory attributes of *Clariid* catfish species of *Clarias gariepinus*,* Heterobranchus bidorsalis*, and their hybrids

**DOI:** 10.1002/fsn3.391

**Published:** 2016-05-25

**Authors:** Wasiu A. Olaniyi, Olukayode A. Makinde, Ofelia G. Omitogun

**Affiliations:** ^1^ Department of Animal ScienceAdekunle Ajasin UniversityPMB 001Akungba‐AkokoOndo State342111Nigeria; ^2^ Department of Animal SciencesObafemi Awolowo UniversityIle‐IfeOsun State220005Nigeria

**Keywords:** *Clariid* catfish, hybrids, sensory attributes, *umami*

## Abstract

*Clariid* catfish are favorite food fish especially in African and Asian continents. Recently there has been preference for particular species or hybrids of these species based on quality assurance and value addition. Consequently, this study aimed to evaluate the possible effect of different catfish species and their hybrids on proximate composition and sensory attributes. Catfish species, *Clarias gariepinus* (CC), *Heterobranchus bidorsalis* (HH), with their hybrid (CH), and reciprocal hybrid (HC) were evaluated for sensory variables – cognitive (sweet, salty, sour, bitter, and recent characteristic taste *‘umami’*) and qualitative (texture, aroma, flavor, and color) tests; and nutritional variables – proximate composition (moisture, protein, ether/fat, and ash). A 5‐point hedonic scale from ‘neutral/neither like nor dislike’ to ‘excellent/like extremely’ was employed in sensory testing. The results showed similar (*P* > 0.05) high moisture contents (>70%) in all species and high but different (*P* < 0.05) ash contents (11–14%) that suggested good sources of mineral elements. The parent species CC and HH had higher ash contents than CH or HC. The crude protein contents were high and similar (*P* > 0.05) across species (>57%). Fat or ether extract was different (*P* < 0.05) and tended to be higher for species with *Clarias* as the female parent than *Heterobranchus*. Sensory analysis showed the parent species, CC and HH, more favorably rated for sweet and *umami* than the hybrids, CH and HC. However, CH was less sour and bitter than all other species and HC better than CH for salty but similar to CC and HH. All fish species were very well liked for texture, but the parent species were superior in flavor than the hybrids. All species were very well liked for aroma, color, and overall acceptability except HC, which was moderately liked. HC rated inferior to the other species overall in sensory attributes. All the fish species did not rate ‘excellent/like extremely’ for any attribute. It can be concluded that the parent catfish species possess better sensory qualities than hybrids, but all species need exogenous enhancement to their natural sensory components.

## Introduction

*Clarias gariepinus* (Burchell, 1822) and *Heterobranchus bidorsalis* (Geoffroy Saint Hilaire, 1809) are of high economic importance in many countries of the world especially African and Asian continents (Legendre et al. 1992; Adebayo and Fagbenro 2004; Olaniyi and Omitogun 2013, 2014); and also serve mainly as food in many homes and hotels (Omitogun et al. 2012). Recently, there has been an increase in the farming of *Clarias* and *Heterobranchus spp* and their hybrids in Nigeria based on their growth performance, short generation interval, and consumer preference or demand, among others. Consequently, there is a high demand for these catfish seeds by fish farmers for stocking.

Interest in hybridization of fish species in aquaculture has been purely for genetic and economic importance, ranging from monosex production to hybrid vigor attributes such as growth performance, robustness, salinity or thermal tolerance, and morphology (Chevassus 1983; Lenormand et al. 1988; Olaniyi and Omitogun 2012).

Nevertheless, the most important attribute of any product regardless of any production technology would appear to be its quality, which is directly dependent on consumer satisfaction or its overall acceptance based on the sensory considerations such as taste, flavor, aroma, and palatability. Generally, sensory attributes of flesh from animals differ from one species to another and even within species, for maturity and sex (Forrest et al. 2001); and the attributes can also vary depending on the chemical composition of products (Heinz and Hautzinger 2007).

Although, *Clarias* and *Heterobranchus spp* and their hybrids are increasingly produced, popular and commonly consumed in Nigeria, there is inadequate or very limited knowledge on comparative nutritional and sensory aspects of their flesh intrinsically important to quality and value‐addition processes. This is underscored by the general observation that the sensory characteristics of catfish flesh is neutral or difficult to distinguish (Fauconneau and Laroche 1996), therefore, the comparison between these fish types will be important to determine whether hybridization affects product nutritional and sensory attributes.

However, the potential challenge of neutrality in sensory characteristics of catfish flesh is on sensory testing. This may require an additional or more discriminating sensory attributes and taste panelists with good sensory capabilities. In the light of this, the study considered an additional taste (5th) to the four primary tastes (sweet, sour, salty, and bitter) in sensory evaluation called *‘umami’. Umami* is a relatively recent characteristic taste imparted by l‐glutamate and 5ˋ‐ribonucleotide compounds such as inosinate and guanylate (Yamaguchi and Ninomiya 2000; Masic and Yeomans 2014), but importantly induced by the presence of monosodium glutamate (Yamamoto 2003; Meilgarrd et al. 2007; Leong et al. 2015). Glutamate is the most abundant amino acid present in many protein‐containing foods such as meat, sea food, and aged cheese (Mouritsen and Styrbaek 2014; Hajeb and Jinap 2015; Kurihara 2015; Leong et al. 2015). *Umami* is expressed as increase in flavor characteristic, continuity, mouthfulness, impact, mildness, and thickness that gives enriching distinctive taste (Yamaguchi and Ninomiya 2000; Leong et al. 2015). Therefore, the general objective of this study was to comparatively evaluate the flesh of *C. gariepinus*,* H. bidorsalis*, and their hybrids for nutritional and sensory attributes and specifically with regard to: (1) the proximate values of these species and their hybrids; (2) cognitive tastes: sweet, salty, sour, bitter, and *umami*; and other qualitative parameters: texture, aroma, flavor, color, and overall acceptability.

## Materials and Methods

### Samples, location, and preparation

The catfish for this study were obtained from the stock held in the Wet laboratory of Department of Animal Sciences, Obafemi Awolowo University, Ile‐Ife, Nigeria. The fish were table‐size (375 ± 33 g) samples from parent species, *Clarias gariepinus* × *Clarias gariepinus* (CC), *Heterobranchus bidorsalis* × *Heterobranchus bidorsalis* (HH), and their hybrids, *C. gariepinus* × *H. bidorsalis* (CH) and reciprocal hybrid, *H. bidorsalis* × *C. gariepinus* (HC).^1^ Only the intestines of the fish samples were eviscerated.

### Proximate evaluation

The nutritional standard methods of the Association of Official Analytical Chemists (AOAC 1990) were followed to determine the moisture, protein, ether extract or fat, and ash contents of each fish sample in triplicates. Moisture content was determined by drying the fresh samples in hot air oven at 70°C to a constant weight; protein by micro‐Kjeldahl method using 6.25 as the conversion factor for total nitrogen to protein; ether extract by the Soxhlet extraction using petroleum spirit; ash determination was carried out in a muffle furnace; and sample burned‐off to remove the organic materials at 600°C for 3 h to a constant weight.

### Cooking

Prior to cooking by boiling, fillet from the fish samples were cut into smaller pieces and rinsed with tap water. The cut parts were then boiled with tap water at 100°C for ~10 min. No condiments were added. After cooking, the fillet were homogenized and served to the taste panel.

### Hedonic scale and quality assessment

The taste evaluation was carried out among catfish eaters or lovers. Selection was made among the tasters to ensure that those with very good taste abilities were picked. Assessment of their sensory prowess was initially established by giving them unidentified common juice to drink and identify. The best 20 trained panelists of equal gender ratio were then chosen for the sensory taste evaluation of the samples. The sensory evaluation of both the cognitive (sweet, salty, sour, bitter, and *umami*) and qualitative parameters (texture, aroma, flavor, color, and overall acceptability) of the catfish species was assessed using a 9‐point hedonic scale (Peryam 1998) adjusted to 5 point according to the following:

5 = Excellent/Like extremely
4 = Very good/Like very much
3 = Good/Like moderately
2 = Fair/Like slightly
1 = Neutral/Neither like nor dislike

**Figure 1:**
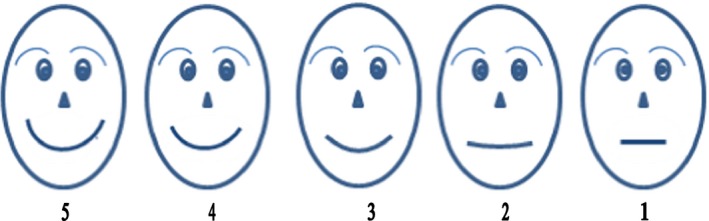
Facial expression in response to stimuli of the varying tastes.

In addition, and congruent to the aforementioned scale, the facial expressions of the tasters were observed in response to the same taste stimuli (Steiner 1987, 1993; Yamaguchi and Ninomiya 2000) (Fig. 1).

### Statistical analysis

Data were arranged in a randomized complete block design with nutrient composition or sensory attributes as main factors and replicates as the second factor, respectively, to increase the precision of the experiment. This is so because effect of replicate is not tested but included to reduce random error, thereby increasing the sensitivity to detect real differences. Consequently, data recorded for proximate composition and hedonic scoring were analyzed with two‐way analysis of variance (ANOVA) using the General Linear Model procedure of SAS (2002) as well as the Duncan's new multiple range test option for separation of means in significantly different treatments (*P* < 0.05).

## Results and Discussion

### Proximate chemical composition

**Figure 2:**
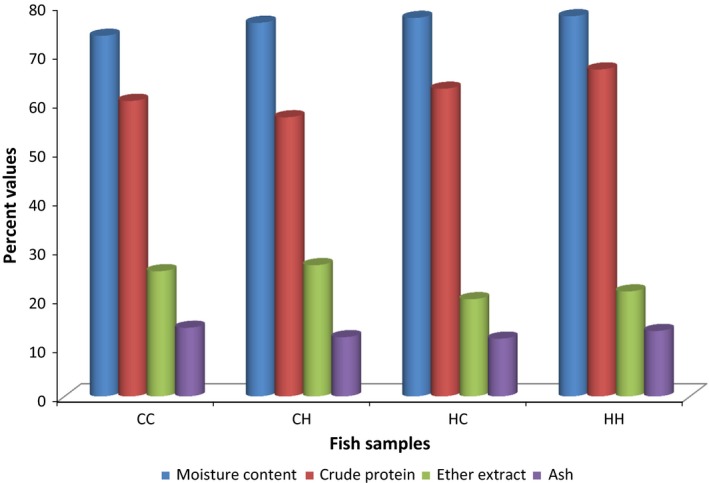
Proximate chemical composition (%) of catfish species of *Clarias gariepinus, Heterobranchus bidorsalis*, and their hybrids. CC, Parent species *Clarias gariepinus* (*Clarias gariepinus* × *Clarias gariepinus*); HH, Parent species *Heterobranchus bidorsalis* (*Heterobranchus bidorsalis* × *Heterobranchus bidorsalis*); CH, Hybrid progeny (*Clarias gariepinus* × *Heterobranchus bidorsalis*); HC, Reciprocal hybrid progeny (*Heterobranchus bidorsalis* × *Clarias gariepinus*).

Table 1 and Figure 2 show the comparison of the catfish species *Clarias gariepinus, Heterobranchus bidorsalis*, and their hybrids for proximate chemical composition. The moisture content was very high in all the fish samples and there were no significant differences among them (*P* > 0.05). However, the moisture content of Hybrid progeny, CH (*Clarias gariepinus* × *Heterobranchus bidorsalis*), was more variable than others (±SD of 12.74 vs. 2.08, 4.16, and 6.03 for CC, HH, and HC, respectively). This may affect ease of processing and uniformity in the final product. Water bound to flesh affects its processing and eating quality (Heinz and Hautzinger 2007). Generally, fish flesh has a high water‐holding capacity compared to other flesh such as meat before slaughtering or processing (Fauconneau et al. 1995; Fauconneau and Laroche 1996; Rosa et al. 2007), but a relatively low water‐holding capacity and a low resistance to mechanical stress (compression, extrusion) when cooked, thus contributing to the juiciness and tenderness of the flesh (Paredes and Baker 1988; Fauconneau et al. 1995; Fauconneau and Laroche 1996). This result is in accordance with earlier studies on fish products (Gall et al. 1983; Gokoglu et al. 2004; Kalogeropoulos et al. 2004; Rosa et al. 2007). The ash content varied significantly (*P* < 0.05) and was generally higher for the parent species, CC and HH, compared to the hybrids species. This may be due to the effects of hybridization. The high values of ash content (11–14%) in this study validates the observation that catfish species are rich in minerals (Sidwell et al. 1978; Oehlenschläger 2000; Ersoy and Yılmaz 2003; Rosa et al. 2007) confirming that they are good mineral sources nutritionally. The ether extract (fat content) varied significantly (*P* < 0.05) between the fish species. The expectation was that there will be tendency for higher fat content for the *Heterobranchus*‐based female parent species than the *Clarias‐*based species due to the deposition of more lipid in the adipose tissue of the *Heterobranchus* parent species (Fauconneau and Laroche 1996) probably due to genetic variability. However, the tendency was lower (*P* < 0.05) values for the *Heterobranchus*‐based species compared to *Clarias*‐based species (HH and HC of 19.88 and 21.47 vs. 25.60 and 26.84 for CC and CH, respectively). This significant variation may be due to total loss or reduction in the adipose tissue when processing *Heterobranchus* samples for analysis. Nevertheless, large differences in lipid content have been reported between many strains (Erickson 1992) and hybrids (Smitherman and Dunham 1983; Fauconneau and Laroche 1996). This may have important bearings on differences in the sensory qualities of the flesh between species because fat has a direct bearing on the taste and flavor of flesh (Heinz and Hautzinger 2007). The crude protein (CP) content was substantial and not significantly different (*P* > 0.05) between species, but more variable for in hybrids than parent species (±SD of 10.87 and 16.05 vs. 1.75 and 2.91 for CH and HC vs. CC and HH, respectively). This may indicate a more stable product quality from the parents than hybrids. The high protein values in this study showed that the catfish species are good sources of valuable proteins.

**Table 1:** Proximate chemical composition of catfish species of *Clarias gariepinus, Heterobranchus bidorsalis*, and their hybrids^1^

Samples (%)	CC	CH	HC	HH
Moisture Content	73.67 ± 2.08	76.33 ± 12.74	77.33 ± 6.03	77.67 ± 4.16
Ash	14.06 ± 8.69^a^	12.13 ± 8.49^b^	11.83 ± 7.73^b^	13.41 ± 9.12^ab^
Ether Extract	25.60 ± 4.93^a^	26.84 ± 1.72^ab^	19.88 ± 6.28^b^	21.47 ± 3.04^ab^
Crude Protein	60.38 ± 1.75	57.05 ± 10.87	62.85 ± 16.05	66.79 ± 2.91

General Linear Model procedure (SAS^®^, 2002).

^a,b^Means ± SD within a row with different superscripts are significantly different (*P* < 0.05).

CC, Parent species *Clarias gariepinus* (*Clarias gariepinus* × *Clarias gariepinus*); HH, Parent species *Heterobranchus bidorsalis* (*Heterobranchus bidorsalis* × *Heterobranchus bidorsalis*); CH, Hybrid progeny (*Clarias gariepinus* × *Heterobranchus bidorsalis*); HC, Reciprocal hybrid progeny (*Heterobranchus bidorsalis* × *Clarias gariepinus*).

Ash, ether extract, and crude protein presented on a dry matter basis.

### Sensory, physical characteristics, and overall acceptability

Table 2 shows the sensory evaluation of both the cognitive (sweet, salty, sour, bitter, and *umami*) and qualitative parameters (texture, aroma, flavor, color, and overall acceptability) of the catfish species. Two of the primary tastes, sweet and salty were not statistically different (*P* > 0.05) among the species, whereas sour, bitter, and *umami* were statistically different (*P* < 0.05). In spite of statistical significance or not, proper interpretation of the hedonic scale rating is based on simple mean scores of preferences or acceptability compared to the scale and mean scores approximated to the nearest whole number (Heinz and Hautzinger 2007). For example, a mean score of 3.5 is approximated to 4 and < 3.5–3. Consequently, the taste attribute of sweet was rated ‘very good/like very much’ for the parent species compared to ‘good/like moderately’ for the hybrids 3.83 and 3.75 compared to 3.42 and 3.12, respectively (Table 2). The sweet‐taste receptors consist of proteins and the process of sensing or detecting sweetness involves the presence of amino acids such as glycine and alanine in the proteins, which bind to the sweet‐taste receptors that are coupled to guanine–nucleotide‐binding proteins (g‐proteins) (Yamamoto 2003). Hence, there is probably presence of more glycine and alanine amino acids in the parent catfish species. Rosa et al. (2007) reported the presence of significant quantities of glycine and alanine in *C. gariepinus*. However, investigation of such amino acid contents of other catfish species may be necessary for further studies. For saltiness, all the species were liked very much except the female parent *Clarias* hybrid (CH), which was liked moderately. The salty nature is usually triggered by the presence of NaCl in samples (Frank 1973; Yamamoto 2003; Chen and Chung 2016). Apart for salty taste being a descriptor, salting is also used in fish processing to improve product shelf life without the danger of toxicity from benzopyrene, cyclic hydrocarbons as found in smoking (Faturoti 1983, 1984; Fauconneau and Laroche 1996). The rating for the sour taste was seemingly opposite to that for salty. The female parent CH was liked very much while the other species were liked moderately. For sour, the higher the rating, the lower the intensity of the taste attribute, this means that CH was less sour than the other species. The taste stimulus responding to hydrochloric acid (HCl) is involved in sour detection compared to other basic stimuli according to labeled‐line theory (Frank 1973; Yamamoto 2003), probably indicating the presence of more HCl‐like cues in CH compared to others. In addition, for all the species, there was nothing like nose wrinkling, lip pursing, and gaping in facial displays, which are attributed to real sour tastes (Yamaguchi and Ninomiya 2000). Similar to sour, the higher the rating for bitterness, the lower the intensity of the taste attribute; which was similar in preferences to sour because the female parent CH was liked very much, whereas the other species were liked moderately. In the same vein, there was no head shaking, frowning, tight closure of the eyes, depressed mouth corners, wide mouth opening and tongue protrusion, wide gaping, spitting, and drooling facial expressions of bitter tastes (Yamaguchi and Ninomiya 2000). These results indicate that both sour and bitter are insignificant in catfish flesh since they were rated good to very good. Furthermore, it would seem as if salty contributes to sour and bitter. In fact, salty, sour, and bitter receptors are closely located on the tongue (Heinz and Hautzinger 2007). The recent characteristic taste, *umami,* recorded highest significant value in the parent species CC and HH than the hybrids (*P* < 0.05) and this was similar to sweet in the pattern of preferences; probably indicating that sweet contributes to *umami*. Indeed, the presence of *umami* taste in products is reportedly triggered by some compounds such as glutamate, a salt of glutamic acid, specific ribonucleotides, and glutamate salts, including monosodium glutamate, potassium glutamate, and calcium glutamate among others (Yamamoto 2003; Meilgarrd et al. 2007; Masic and Yeomans 2014; Hajeb and Jinap 2015; Leong et al. 2015; Chen and Chung 2016). The basic taste cues of glutamate is its sweetness, therefore, the positive evaluation of *umami* taste in this study probably indicates the presence of one or more of these compounds that triggered its taste receptors. Efeyan et al. (2015) highlighted the T1R family members of G‐protein–coupled receptors being responsible for the detection of *umami* taste. Rosa et al. (2007) reported the presence of glutamic acid in *C. gariepinus* products; therefore, further tests may be necessary to quantify glutamate content in other catfish species and their hybrids for nutritional purposes. The qualitative properties of aroma, flavor, texture, and color showed no statistically significant differences (*P* > 0.05) between fish species (Table 2). The texture was liked very much across all species probably because of a similar relatively low water‐holding capacity and a low resistance to mechanical stress (compression, extrusion) when cooked, thus contributing to the juiciness and tenderness of the flesh (Paredes and Baker 1988; Fauconneau et al. 1995; Fauconneau and Laroche 1996). Aroma and color were liked very much by all species except the female *Heterobranchus* hybrid (HC), which was liked moderately on both counts. However, it has been reported that the aroma and color may be affected by fatty acid composition and resultant effects of lipid oxidation (Heaton et al. 1973; Fauconneau and Laroche 1996; Heinz and Hautzinger 2007). Genetic differences due to hybridization may be responsible for this observation. In relation to this, the flavor of the parents was better rated than those of the hybrids, probably indicating a masking effect due to hybridization. Flavor comprises taste and smell, which are directly impacted by fat type and content (Heinz and Hautzinger 2007) and large differences in lipid content of catfish have been reported between many strains (Erickson 1992) and hybrids (Smitherman and Dunham 1983; Fauconneau and Laroche 1996). The overall acceptability or palatability rating showed a reflection or aggregation of the ratings for all cognitive and qualitative sensory attributes. The data revealed statistically significant differences (*P* < 0.05) between the species in the overall acceptability, with the parent species and the hybrid, CH very well liked more than HC, which generally followed the trend of all ratings. However, none of the attributes was rated excellent or liked extremely, probably indicating some deficiencies in the sensory attributes of the catfish species studied.

**Table 2:** Sensory evaluation and acceptability of catfish species of *Clarias gariepinus, Heterobranchus bidorsalis*, and their hybrids

Parameter	CC	CH	HC	HH
Sweet	3.83 ± 1.03	3.42 ± 1.08	3.12 ± 1.19	3.75 ± 1.14
Sour	3.00 ± 1.41^ab^	3.50 ± 1.38^a^	3.00 ± 1.28^ab^	2.75 ± 1.29^b^
Bitter	2.83 ± 1.53^b^	3.58 ± 1.68^a^	3.00 ± 1.35^b^	3.00 ± 1.48^ab^
Salty	3.67 ± 1.44	3.42 ± 1.56	3.50 ± 1.45	3.67 ± 1.37
*Umami*	3.75 ± 1.36^ab^	3.42 ± 1.17^b^	3.00 ± 1.21^b^	4.42 ± 0.90^a^
Texture	3.67 ± 1.30	3.67 ± 1.23	3.58 ± 0.90	4.00 ± 0.95
Aroma	3.50 ± 1.38	3.50 ± 1.24	3.25 ± 1.36	3.92 ± 1.24
Flavor	4.08 ± 1.17	3.33 ± 1.37	3.25 ± 1.06	3.83 ± 1.12
Color	4.00 ± 1.04	3.50 ± 1.51	3.33 ± 1.30	4.00 ± 1.21
Overall acceptability	4.00 ± 1.00^a^	3.55 ± 0.69^ab^	2.72 ± 1.10^b^	4.00 ± 1.10^a^

^ab^Means within a row with different superscripts are significantly different (*P* < 0.05).

General Linear Model procedure (SAS^®^, 2002).

CC, Parent species *Clarias gariepinus* (*Clarias gariepinus* × *Clarias gariepinus*); HH, Parent species *Heterobranchus bidorsalis* (*Heterobranchus bidorsalis* × *Heterobranchus bidorsalis*); CH, Hybrid progeny (*Clarias gariepinus* × *Heterobranchus bidorsalis*); HC, Reciprocal hybrid progeny (*Heterobranchus bidorsalis* × *Clarias gariepinus*).

## Conclusion

The study revealed that catfish species of *C. gariepinus, H. bidorsalis*, and their hybrids are good sources of protein, fat, and mineral contents that are indispensable in human diets and essential for nutritional regimen. The sensory analyses, which determined the overall acceptability/palatability showed almost similar and favorable preferences for all the products except the reciprocal hybrid HC. The positive evaluation of *umami* taste probably indicated the presence of l‐glutamate and 5ˋ‐ribonucleotides compounds in the catfish species studied, and its distinctive rich taste has really contributed to the sweetness as expressed in the sensory attributes. However, additional condiments may be added to the products during processing to enhance the natural taste or palatability components in the fish.

## Conflict of Interest

None declared.

## Note

1 The female parent species are mentioned first in the species breeding.
